# Supplementary material for: A New Stilbene Glucoside from Biotransformation-Guided Purification of Chinese Herb Ha-Soo-Oh
Source: Plants (Basel). 2022 Sep 1;11(17):2286. doi: 10.3390/plants11172286 (PMC9460137; doi:10.3390/plants11172286)
Supplement: Supplementary file 1 [file plants-11-02286-s001.zip › plants-1869958-supplementary.pdf]

Supplemental Materials

A New Stilbene Glucoside from Biotransformation-Guided Purification of Chinese Herb Ha-Soo-Oh

Jiumn-Yih Wu [1,†], Hsiou-Yu Ding [2,†], Tzi-Yuan Wang [3,†], Min-Hui Hsu [4] and Te-Sheng Chang [4,*]

[1] Department of Food Science, National Quemoy University, Kinmen County 892, Taiwan
[2] Department of Cosmetic Science, Chia Nan University of Pharmacy and Science, No. 60 Erh-Jen Rd., Sec. 1, Tainan 71710, Taiwan
[3] Biodiversity Research Center, Academia Sinica, Taipei 11529, Taiwan
[4] Department of Biological Sciences and Technology, National University of Tainan, Tainan 70005, Taiwan
* Correspondence: mozyme2001@gmail.com; Tel./Fax: +886-6-2602137
† These authors contributed equally to this work.

Contents

Table S1. ^{13}C- and ^{1}H-NMR spectral data and HMBC, NOESY correlations of compounds (**1**)[a]. (DMSO-d_6)

Figure S1. The high-resolution mass analysis of PSG (**1**) at the negative mode. A significant signal at *m/z* 421.1129.

Figure S2. ^{1}H-NMR spectrum (700 MHz, DMSO-d_6) of the PSG (**1**).

Figure S3. ^{13}C-NMR spectrum (175 MHz, DMSO- d_6) of the PSG (**1**).

Figure S4. DEPT-NMR spectrum (175 MHz, DMSO-d_6) of the PSG (**1**).

Figure S5. ^{1}H-^{13}C HSQC-NMR spectrum (700 MHz, DMSO-d_6) of the PSG (**1**).

Figure S6. ^{1}H-^{13}C HMBC-NMR spectrum (700 MHz, DMSO-d_6) of the PSG (**1**).

Figure S7. ^{1}H-^{1}H COSY-NMR spectrum (700 MHz, DMSO-d_6) of the PSG (**1**).

Figure S8. ^{1}H-^{1}H NOESY-NMR spectrum (700 MHz, DMSO-d_6) of the PSG (**1**).

Figure S9. The high-resolution mass analysis of TSG at the negative mode. A significant signal at *m/z* 405.1180.

Table S1. ^{13}C- and ^{1}H-NMR spectral data and HMBC, NOESY correlations of compounds (**1**)[a]. (DMSO-d_6)

position	δ_C	δ_H (δ in ppm, *J* in Hz)	HMBC	NOESY
1	129.1		H-7, 8	
2	136.3		H-4, 6, 7, 1''	
3	150.5		H-4	
4	102.6	6.17 (d, *J*=2.8)	H-6	
5	154.6		H-4, 6	
6	101.0	6.53 (d, *J*=2.8)	H-4, 7	H-6/H-8
7	120.4	7.61 (d, *J*=16.5)	H-6, 8	H-7/H-8, 2', 6', 1''
8	131.9	6.80 (d, *J*=16.5)		H-8/H-6, 7, 2'
1'	128.7		H-7, 2', 5', 6'	
2'	113.8	7.00 (d, *J*=2.1)	H-8, 6'	H-2'/H-7, 8, 2''
3'	145.2		H-2', 5'	
4'	145.4		H-2', 5', 6'	
5'	115.7	6.70 (d, *J*=7.7)	H-6'	H-5'/ H-6', 2''
6'	118.6	6.90 (dd, *J*=2.1, 7.7)	H-8, 2'	H-6'/H7, 5', 2'', 6b''
1''	106.5	4.41 (d, *J*=7.7)	H-2'', 3'', 5''	H-1''/ H-3'', 5''
2''	74.0	3.36, (m)	H-1'', 3'', 4''	H-2''/ H-4''
3''	76.1	3.28, (m)[b]	H-1'', 2'', 4''	H-3''/H-1'', 5''
4''	69.4	3.26, (m)[b]	H-3'', 5'', 6b''	H-4''/ H-2'', 6a''
5''	77.1	3.22, (m)	H-1'', 4'', 6a''	H-5''/ H-1'', 6b''
6''	60.7	3.55, (m)	H- 4''	H-6a''/ H-4'', 6b''
		3.68, (m)		H-6b''/ H-5'', 6a''

[a] ^{1}H NMR data was determined on 700 MHz spectrometer and ^{13}C NMR data on 175 MHz spectrometer.

[b] Overlapping

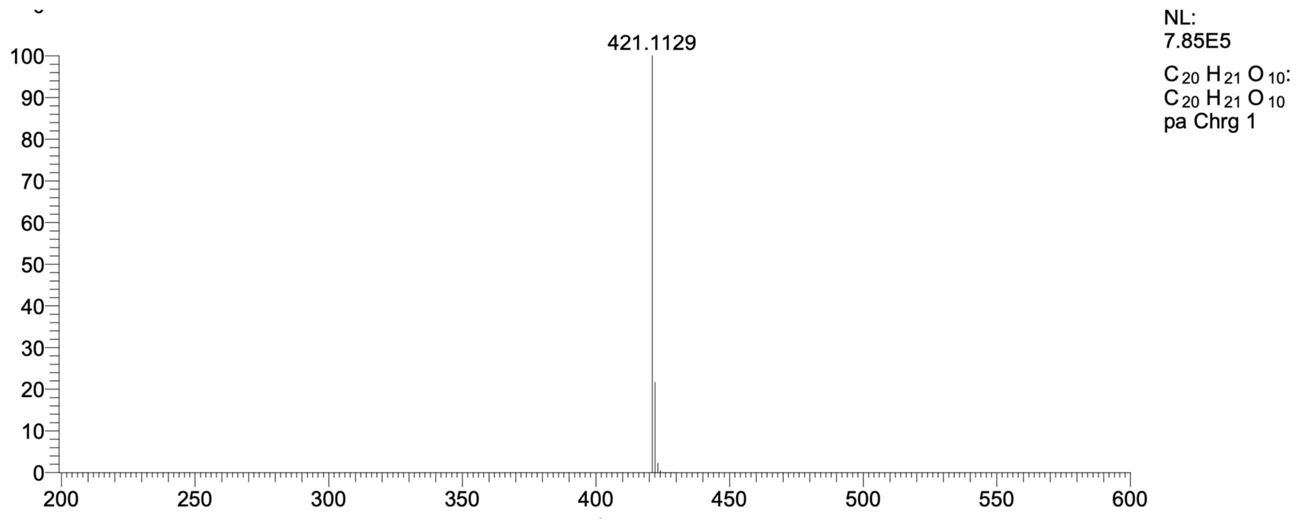

Figure S1. The high-resolution mass analysis of PSG (**1**) at the negative mode. A significant signal at *m*/*z* 421.1129.

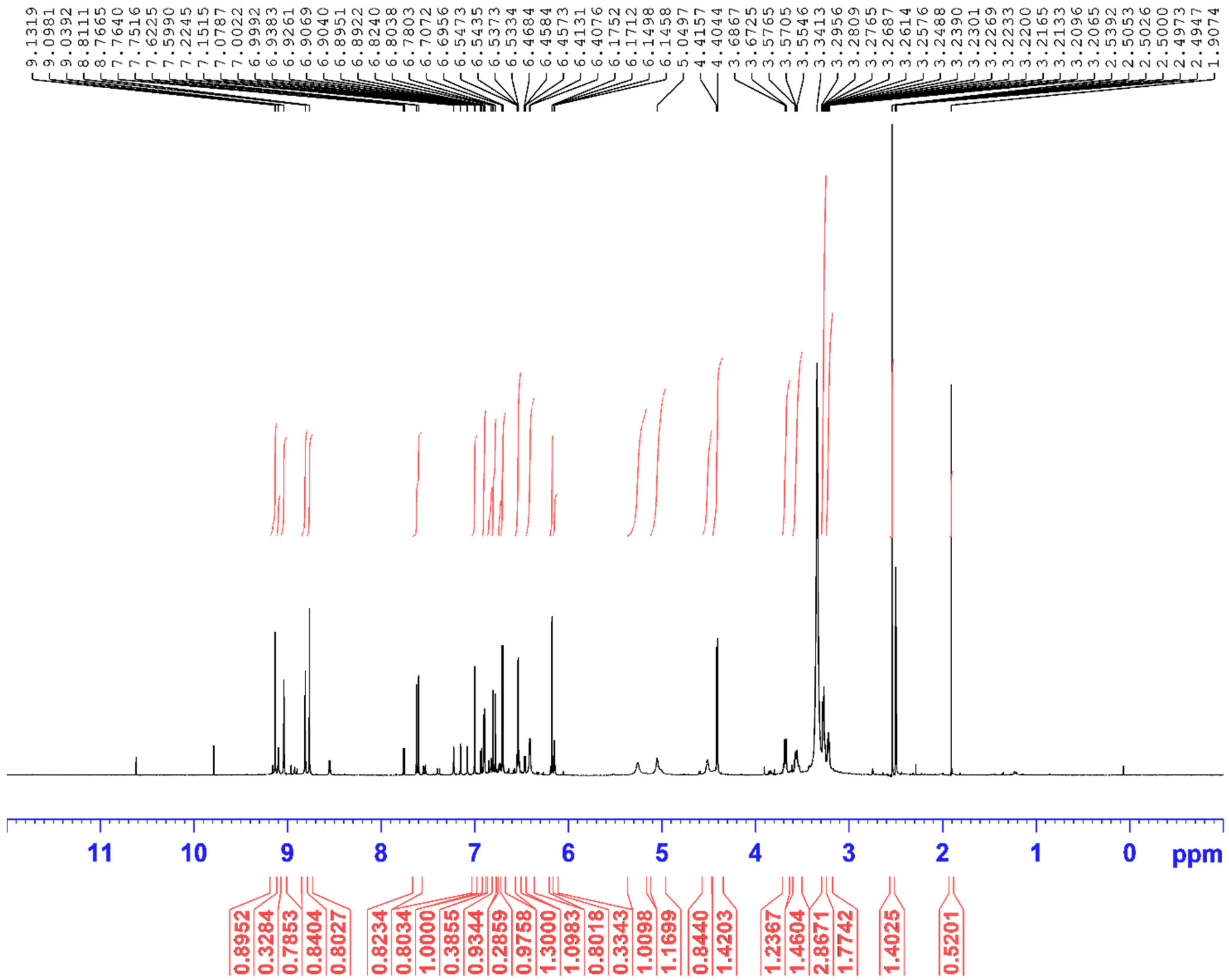

Figure S2. ^{1}H-NMR spectrum (700 MHz, DMSO-d_6) of the PSG (**1**).

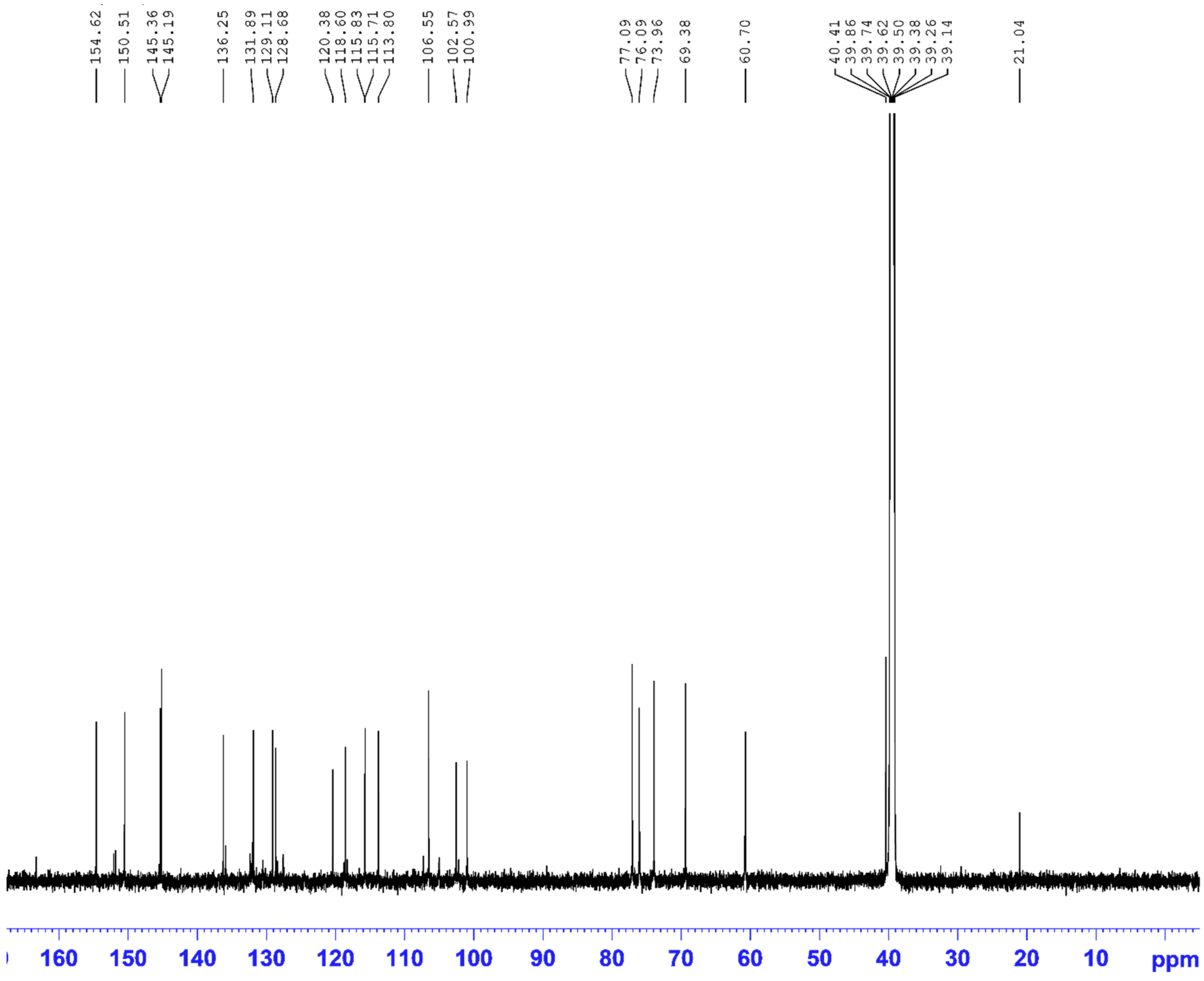

Figure S3. ^{13}C-NMR spectrum (175 MHz, DMSO- d_6) of the PSG (**1**).

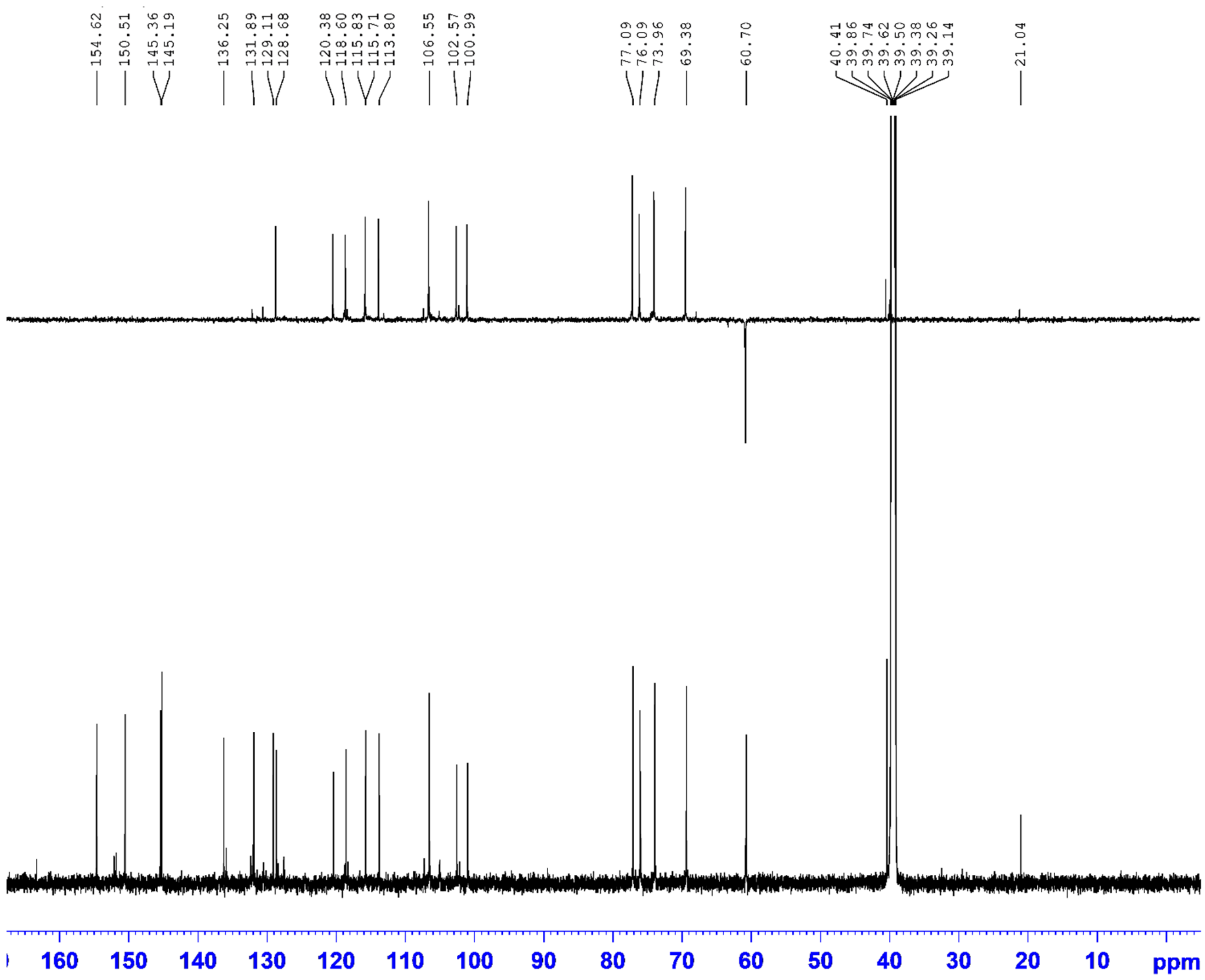

Figure S4. DEPT-NMR spectrum (175 MHz, DMSO-d_6) of the PSG (**1**).

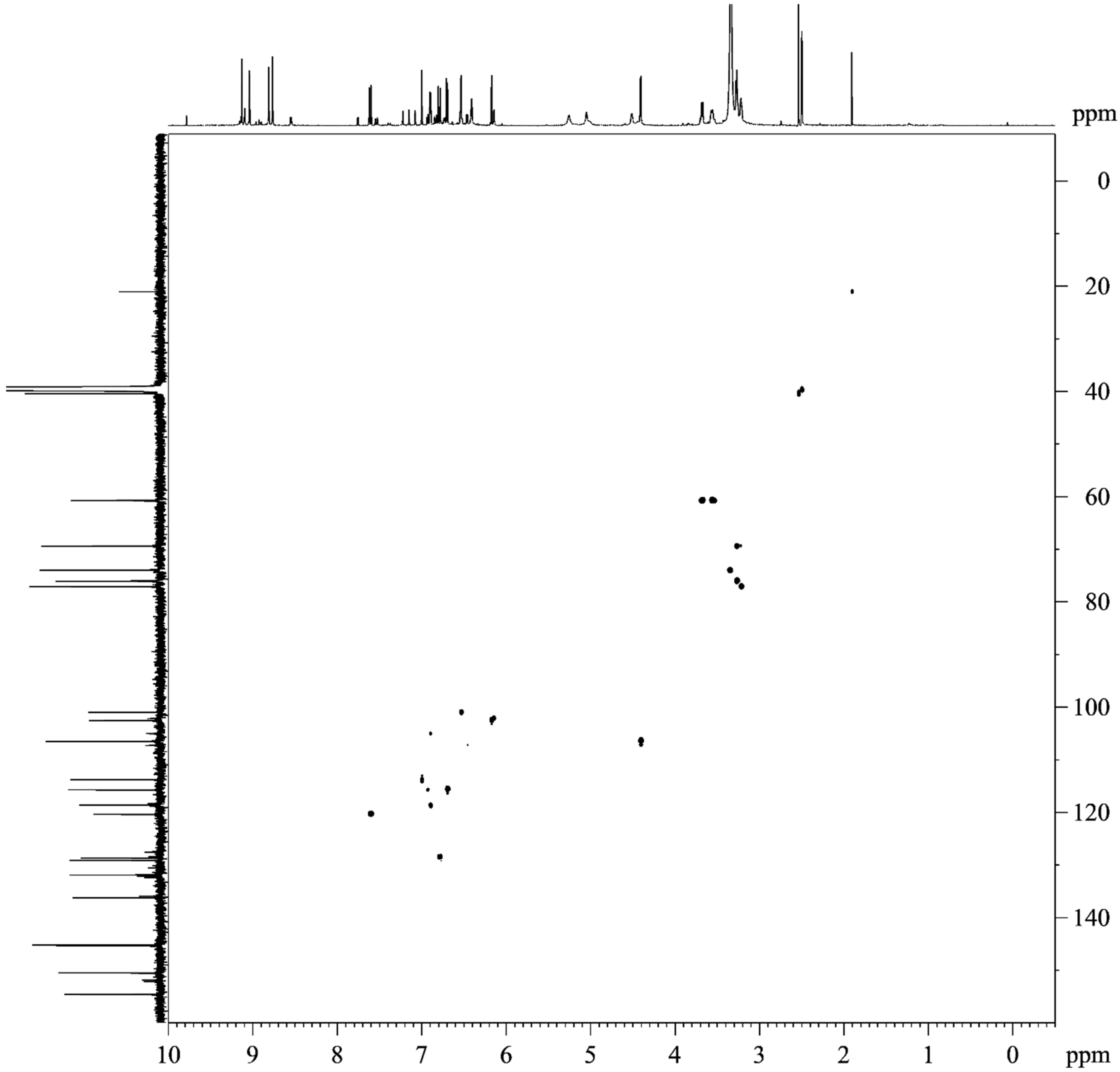

Figure S5. ^{1}H-^{13}C HSQC-NMR spectrum (700 MHz, DMSO-d_6) of the PSG (**1**).

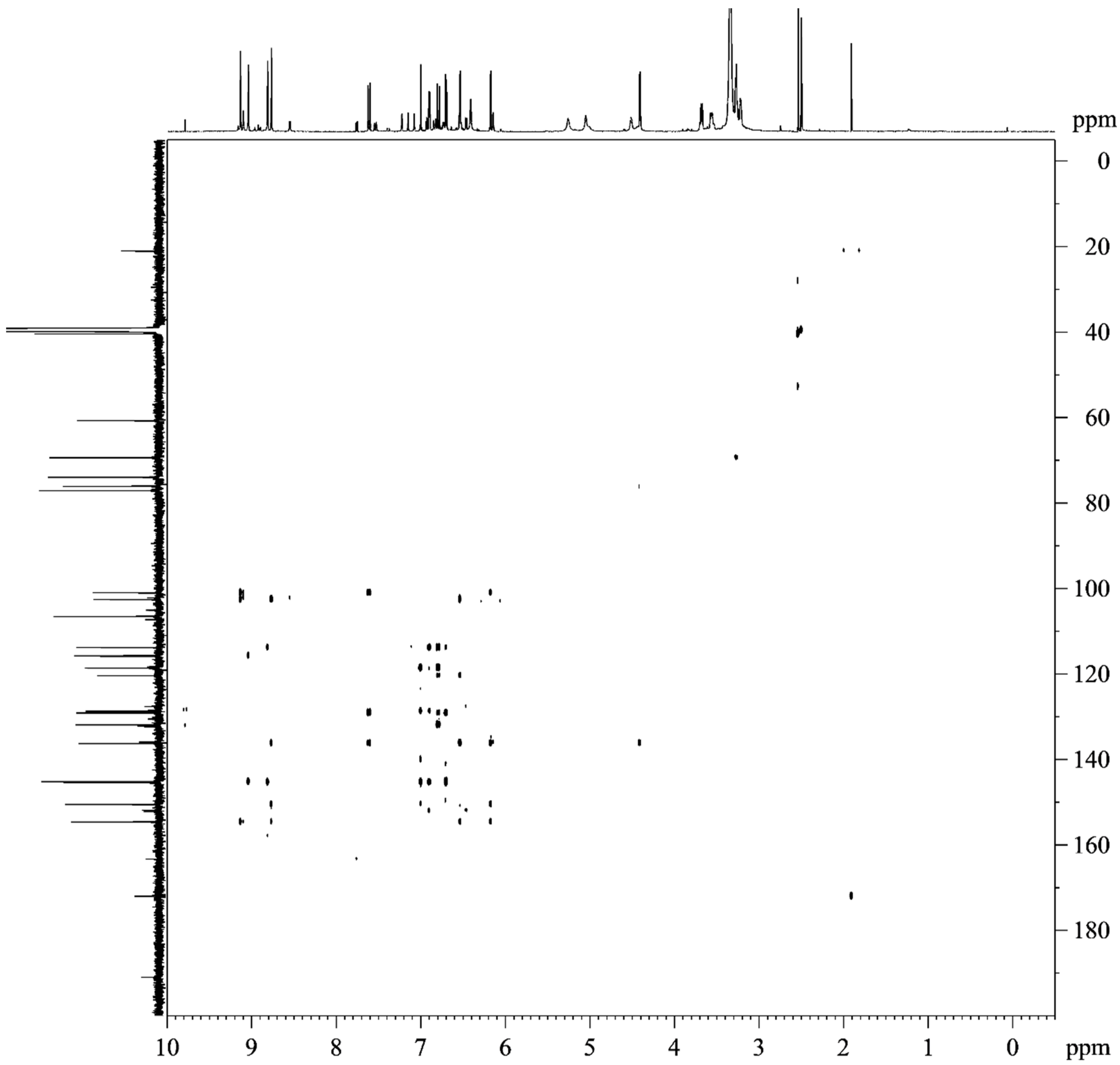

Figure S6. ^{1}H-^{13}C HMBC-NMR spectrum (700 MHz, DMSO-d_6) of the PSG (**1**).

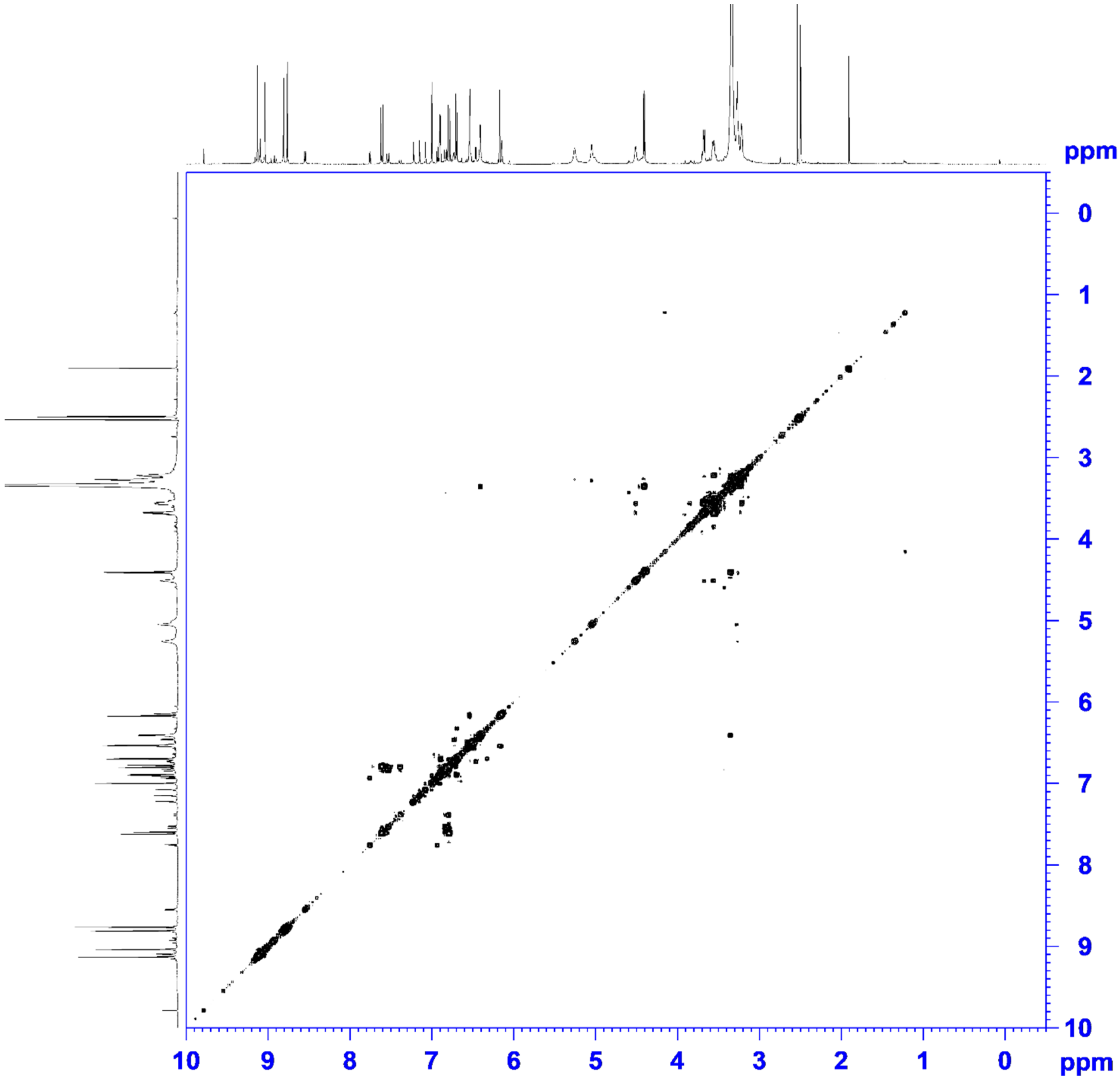

Figure S7. ^{1}H-^{1}H COSY-NMR spectrum (700 MHz, DMSO-*d*$_6$) of the PSG (**1**).

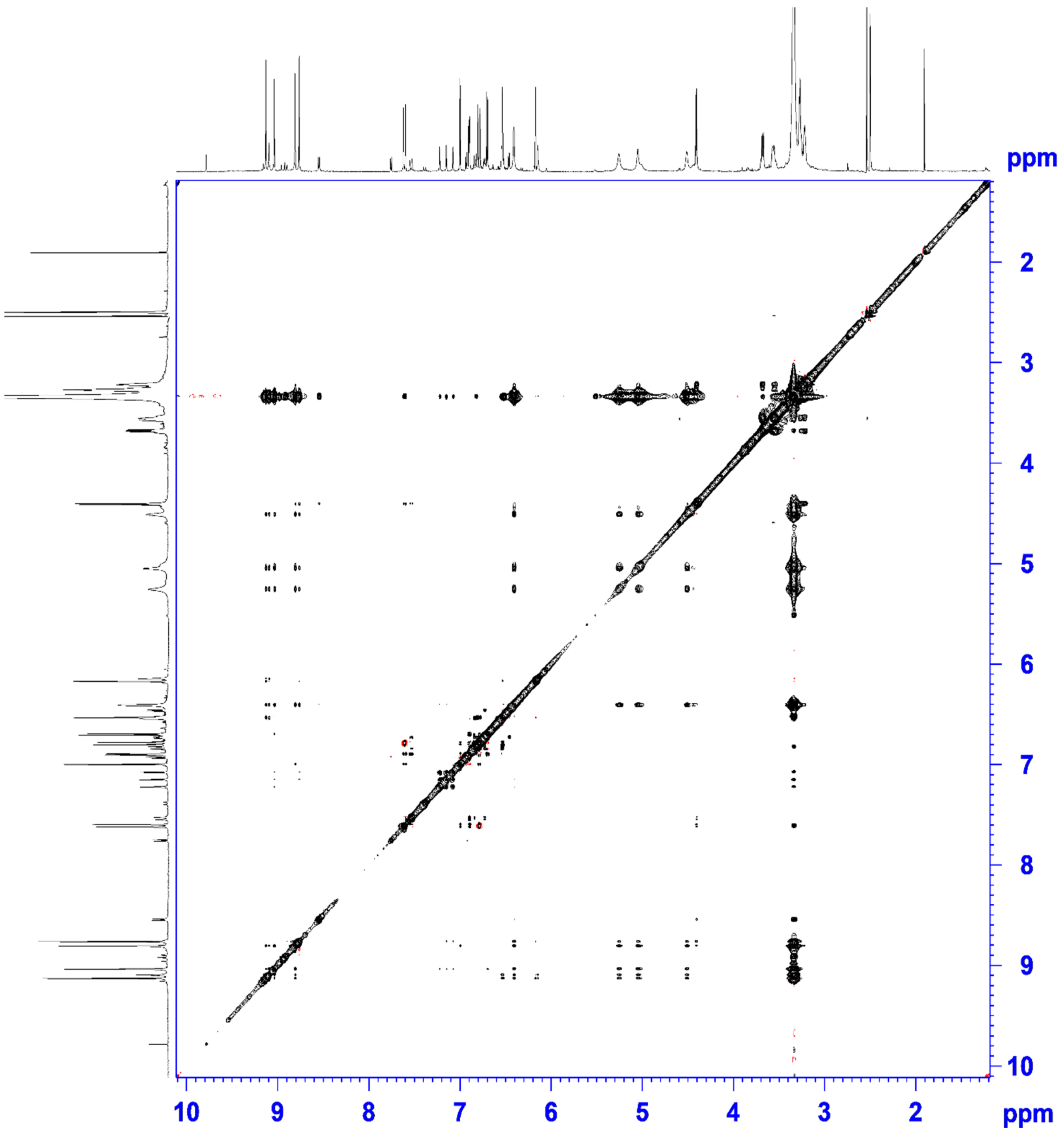

Figure S8. ^{1}H-^{1}H NOESY-NMR spectrum (700 MHz, DMSO-d_6) of the PSG (**1**).

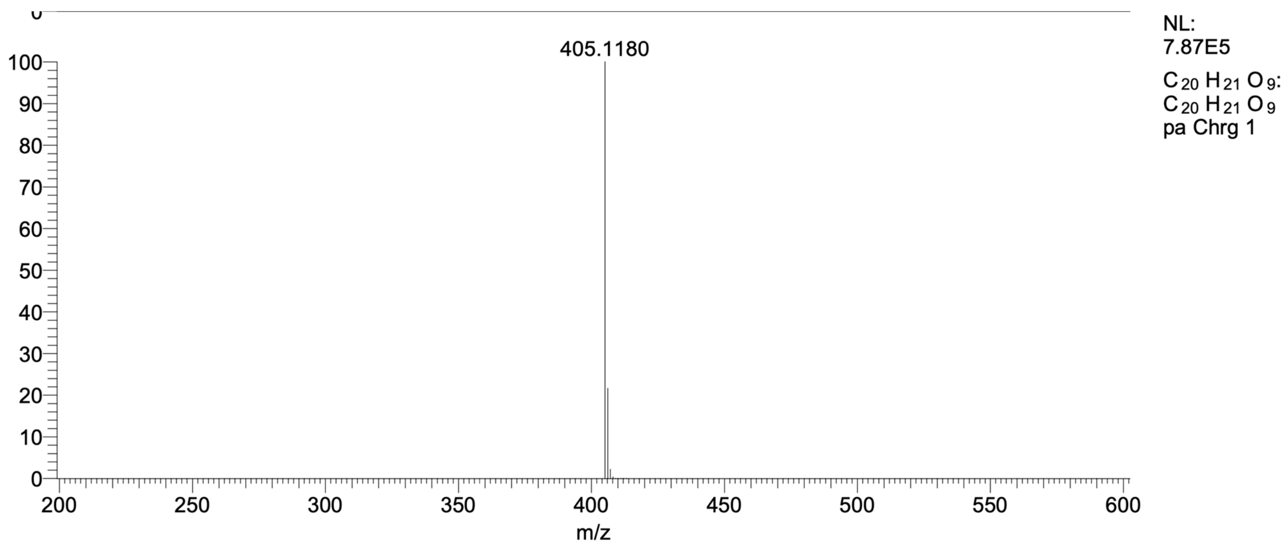

Figure S9. The high-resolution mass analysis of TSG at the negative mode. A significant signal at *m/z* 405.1180.
